# Unfolding rates of 1:1 and 2:1 complex of CX-5461 and c-*MYC* promoter G-quadruplexes revealed by single-molecule force spectroscopy

**DOI:** 10.52601/bpr.2024.240018

**Published:** 2024-06-30

**Authors:** Hui Peng, Yashuo Zhang, Qun Luo, Xinyu Wang, Huijuan You

**Affiliations:** 1 Hubei Key Laboratory of Natural Medicinal Chemistry and Resource Evaluation, School of Pharmacy, Tongji Medical College, Huazhong University of Science and Technology, Wuhan 430030, China; 2 College of Physics Science and Technology, Yangzhou University, Yangzhou 225009, China

**Keywords:** Single-molecule, G-quadruplex, Magnetic tweezers, Stoichiometry, Force spectroscopy, Pidnarulex

## Abstract

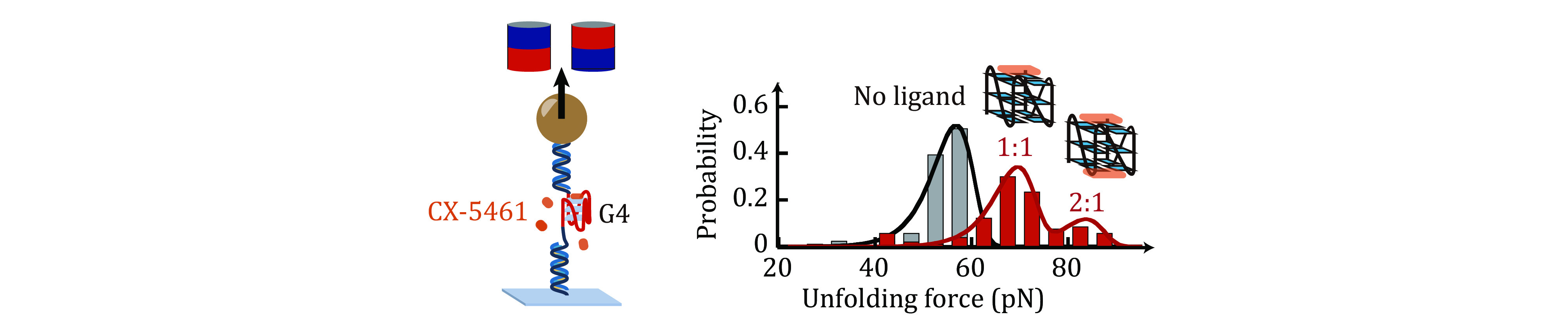

CX-5461, also known as pidnarulex, is a strong G4 stabilizer and has received FDA fast-track designation for BRCA1- and BRCA2- mutated cancers. However, quantitative measurements of the unfolding rates of CX-5461-G4 complexes which are important for the regulation function of G4s, remain lacking. Here, we employ single-molecule magnetic tweezers to measure the unfolding force distributions of c-*MYC* G4s in the presence of different concentrations of CX-5461. The unfolding force distributions exhibit three discrete levels of unfolding force peaks, corresponding to three binding modes. In combination with a fluorescent quenching assay and molecular docking to previously reported ligand-c-*MYC* G4 structure, we assigned the ~69 pN peak corresponding to the 1:1 (ligand:G4) complex where CX-5461 binds at the G4’s 5'-end. The ~84 pN peak is attributed to the 2:1 complex where CX-5461 occupies both the 5' and 3'. Furthermore, using the Bell-Arrhenius model to fit the unfolding force distributions, we determined the zero-force unfolding rates of 1:1, and 2:1 complexes to be (2.4 ± 0.9) × 10^−8^ s^−1^ and (1.4 ± 1.0) × 10^−9^ s^−1^ respectively. These findings provide valuable insights for the development of G4-targeted ligands to combat c-*MYC*-driven cancers.

## INTRODUCTION

G-quadruplexes (G4s) in oncogene promoters have emerged as promising targets for both regulating gene expression (Balasubramanian *et al.* 2011) and developing cancer therapies (Wang and Vasquez 2023). Chromatin immunoprecipitation sequencing (ChIP-seq) experiments using a G4-specific antibody revealed over 123,000 G4 structures within human chromatin (Hänsel-Hertsch *et al.* 2016). Notably, G4 structures present in >60% of promoters and ~70% of genes, with particular enrichment in cancer-related genes and regions with somatic copy number amplifications, such as c-*MYC* (Hänsel-Hertsch *et al.* 2016; Lago *et al.* 2021; Zheng *et al.* 2020). c-*MYC*, a proto-oncogene with transcription factor activity, plays a critical role in cancer progression when its expression is unregulated, especially due to mutation (Chaudhuri *et al.* 2021; Neidle 2017). The c-*MYC* gene has two promoters, with the P1 promoter, containing a crucial region called nuclease hypersensitive element III (NHE III) (1). This NHE III region controls 80%–95% of the c-*MYC* gene’s transcriptional activity (Chaudhuri *et al.* 2021). Pioneering work by the Hurley group demonstrated that small molecules stabilizing G4 structures can inhibit c-*MYC* transcription, highlighting G4s as a promising anti-cancer target (Siddiqui-Jain *et al.* 2002). To date, over 4300 different G4-targeted ligands have been identified according to the G4 targeting ligand database G4LDB 2.2 (http://www.g4ldb.com) (Asamitsu *et al.* 2019; Wang *et al.* 2022). These ligands include diverse chemical classes such as 2,6-diamido anthraquinones (Sun *et al.* 1997), porphyrin derivative TmPyP4 (Siddiqui-Jain *et al.* 2002), perylene derivative PIPER (Rangan *et al.* 2001), acridine derivative BRACO-19 (Moore *et al.* 2006) and quinoline derivative pyridostatin (PDS) (Rodriguez *et al.* 2008).

CX-5461 (pidnarulex), a naphthyridine derivative, was initially developed as an RNA polymerase I transcription inhibitor for the treatment of hematological malignancies (Khot *et al.* 2019). Recent studies have revealed that the mechanism underpinning the therapeutic efficacy of CX-5461 includes stabilizing G-quadruplex formation and inhibiting topoisomerase II, causing DNA damage (Bossaert *et al.* 2021; Bruno *et al.* 2020; Koh *et al.* 2024; Pan *et al.* 2021). The phase I clinical trial of CX-5461 (NCT02719977) in homologous recombination-deficient solid tumor established clinical proof-of-concept for G4s stabilizer (Hilton *et al.* 2022). However, the kinetic basis of G4s stabilization by CX-5461 remains not fully understood.

Single-molecule force spectroscopy (SMFS) is a powerful tool for investigating the folding and unfolding kinetics of c-*MYC* promoter G4s under physiologically relevant conditions (Cheng *et al.* 2020; You *et al.* 2015). This technique allows us to quantify how proteins (Cheng *et al.* 2021; You *et al.* 2017) and small ligands (Zhang *et al.* 2024) influence the folding/unfolding rates of c-*MYC* G4s. We recently employed SMFS with magnetic tweezers and demonstrated that CX-5461 is a strong G4s stabilizer, which significantly increases the average unfolding forces of c-MYC G4s at physiologically relevant potassium concentrations (Zhang *et al.* 2024). While average unfolding forces provide valuable information about the G4s stabilization, they don’t quantify the unfolding rates of CX-5461-G4 complexes. This highlights the untapped potential of SMFS for a deeper understanding of ligand−G4 interactions.

Importantly, ligands can bind to G4s in various stoichiometries (binding ratios). Previous studies have utilized mass spectrometry (MS) to quantify the stoichiometry of several important ligands with telomeric G4s, revealing 1:1 or 2:1 (ligands:G4s) complexes (Marchand *et al.* 2018). Similarly, MS analysis identified imidazole derivatives forming 1:1, 2:1, and 3:1 complexes with c-*MYC* G4s (Li *et al.* 2022). NMR structural studies further revealed that the carbazole compound BMVC initially binds the 5'-end of c-MYC G4s in a 1:1 complex, and at higher ratios, also binds the 3'-end to form a 2:1 complex (Liu *et al.* 2019). NMR and crystal structures reveal that ligand binding to G4s relies on π–π stacking interactions between the ligands’ hetero-aromatic moieties and the planar G-quartets at the 5'- or 3'-ends of G4s (Dickerhoff *et al.* 2021a; Liu *et al.* 2019). However, the impact of different binding stoichiometries (1:1 or 2:1) on the G4’s unfolding rates and unfolding energy barriers remains unclear.

**Figure 1:**
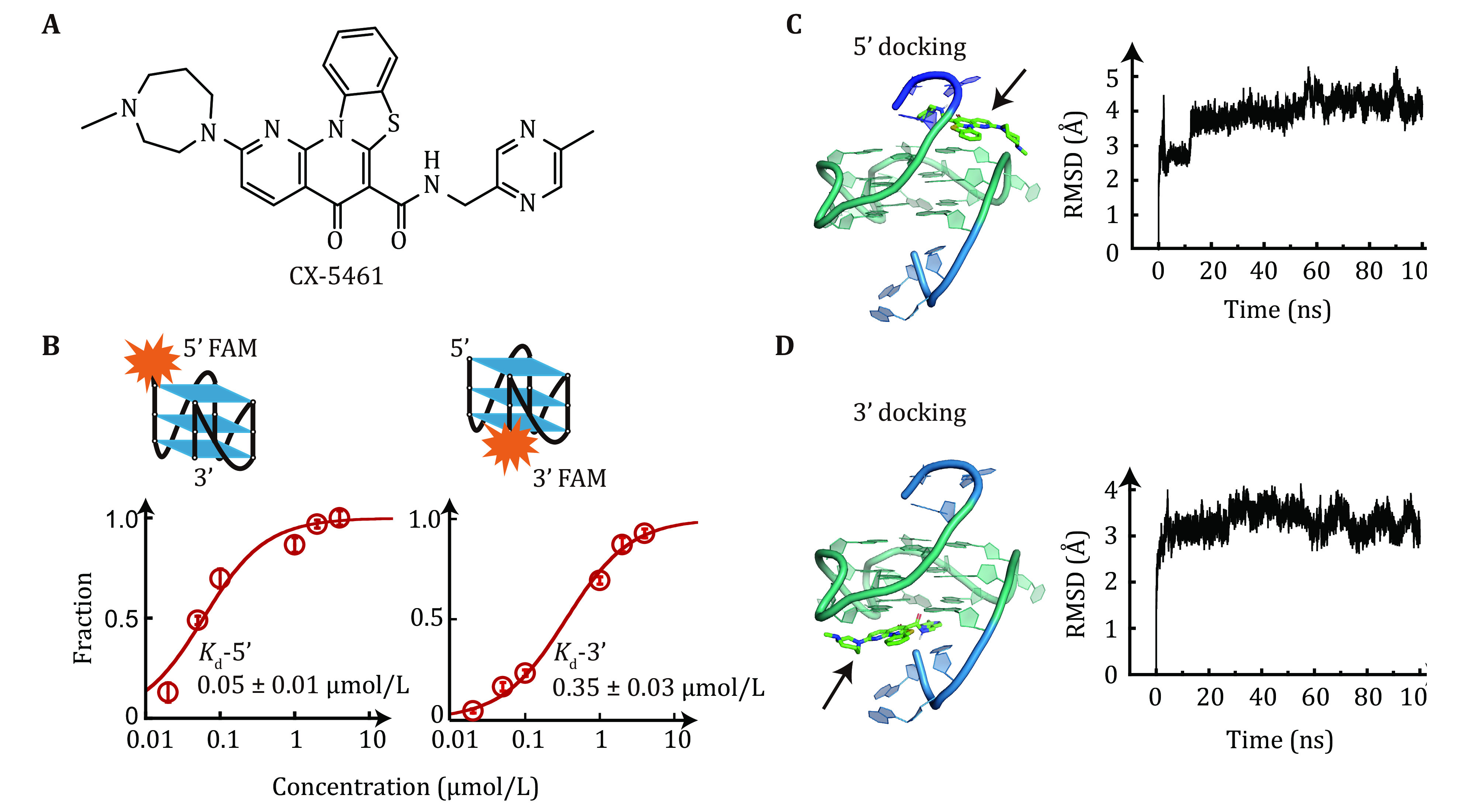
Binding affinity of CX-5461 to c-*MYC* G4s. **A** Chemical structure of CX-5461. CX-5461 contains a rigid benzothiazole-based core region and two flexible side chains. **B** Dissociation constant *K*_d_ of CX-5461 binding to 5'-FAM-MYC G4 or 3'-FAM-MYC G4 determined by fluorescence quenching assay. The data points represent the average of three replicates, with error bars indicating the standard error. **C**,**D** Molecular docking simulations. Left panel: the lowest-energy binding conformations of CX-5461 docked to the 5'-end (**C**) and 3'-end of G4s (**D**). The BMVC c-*MYC* G4 complex (PDB ID:6O2L) was used for semi-flexible global docking. Right panel: root means square deviation (RMSD) of the CX-5461-G4 complexes during a 100 ns molecular dynamics simulation. The RMSD values remained below 0.5 nm, suggesting the initial docking poses were valid

Here, using our recently developed single-molecule manipulation methods (Zhang *et al.* 2024), we conducted further measurements of the unfolding-force distributions of c-*MYC* G4s in the presence of different concentrations of CX-5461 (Fig. 1A). By combining these data with the binding affinities of CX-5461 to both 5'- and 3'-end of c-*MYC* G4s, determined through a fluorescent quenching assay, we were able to assign the observed unfolding force peaks to distinct CX-5461-G4 complexes (1:1 and 2:1 stoichiometry). This approach allowed us to estimate the estimated zero-force unfolding rates of 1:1 and 2:1 CX-5461-*MYC* G4 complexes.

## RESULTS

### CX-5461 binding affinity to 5'- and 3'-end of c-*MYC* G4 measured by fluorescence quenching assay

To elucidate the binding affinity of CX-5461 to c-*MYC* G4s, we employed a fluorescence quenching assay. The single-stranded DNA (ssDNA) c-*MYC* G4s forming sequence was labeled with a carboxyfluorescein (FAM) fluorophore at either the 5'-end or 3'-end and titrated with different concentrations of CX-5461. CX-5461 does not directly quench the fluorescein dye at a concentration of 1 μmol/L (Zhang *et al.* 2024), but upon binds to c-*MYC* G4s, CX-5461 induced quenching of FAM fluorescence in both 5' and 3' FAM-labeled c-*MYC* G4s, indicating its ability to bind at both ends. Thus, the dissociation constant *K*_d_ of CX-5461 to the binding site on each end of c-*MYC* G4s can be determined by analyzing the changes in fluorescence intensity. The data was fitted with a quadratic equation, and we obtained the *K*_d_ value for the 5'-FAM G4s (0.05 ± 0.01 μmol/L, average ± standard error) was significantly lower compared to the 3'-FAM G4s (0.35 ± 0.03 μmol/L, average ± standard error) (Fig. 1B). This finding indicated that CX-5461 binds more strongly to the 5'-end than 3'-end of c-MYC G4s. The higher binding affinity of G4s ligands to 5'-end than 3'-end of c-MYC G4s was also observed for several other G4s targeting ligands (Li *et al.* 2022). The binding energy of G4s can be calculated according to the equilibrium constant $ \Delta G={k}_{B}T\mathrm{ln}{K}_{d} $, where $ {k}_{B} $ is the Boltzmann constant and *T* is the absolute temperature. CX-5461 binding the 5'-end and 3'-end complexes were estimated as $ \Delta {G}_{{\mathrm{binding}}-5\text{'}{\mathrm{end}}} $ = –16.8*k*_B_*T*, corresponding to –9.95 kcal/mol, and $ \Delta {G}_{{\mathrm{binding}}-3\text{'}{\mathrm{end}}} $ = –14.9*k*_B_*T*, which corresponds to –8.82 kcal/mol.

To elucidate the binding modes of CX-5461 with c-*MYC* G4s, we performed a molecular docking simulation using the previously reported solution structure of the 2:1 ligand-G4s complexes (Liu *et al.* 2019). Figure 1C depicts the lowest energy binding conformation of CX-5461, where it stacks on the 5'-end of the c-*MYC* G4s with the docking score of –10 kcal/mol. This docked conformation reveals that the rigid benzothiazole rings of CX-5461 engage in π–π stacking interactions with two guanine bases, while the two flexible side chains interact with the 5' terminal residues and the phosphate backbone through electrostatic and hydrogen-bonding interactions. Figure 1D shows the lowest docking conformation of CX-5461 bound to the 3'-end of c-*MYC* G4s with a docking score of –9.3 kcal/mol. The docking results revealed a strong preference for 5’-end binding conformations. Among 100 docked conformations, 92 conformations showed binding at the 5'-end with the lowest docking score of –10 kcal/mol. Conversely, only seven conformations bound to the 3'-end of G4s. We further performed molecular dynamics simulation using both 5'-end and 3'-end docked conformations as initial structures. The root-mean-square deviation (RMSD) remained within 5 Å, suggesting the validity of the initial docking poses (Figs. 1C and 1D). It is important to note that while docking scores provide relative binding affinities, they don’t directly refer to the ligand binding energy. A systematic evaluation using different docking programs to analyze different G4s–ligand interactions showed that docking accuracy is limited by the scoring function (Dickerhoff *et al.* 2021b). Despite limitations, four ligands (quindoline I, BMVC, PEQ, and DC4) bind to parallel-stranded c-*MYC* G4s in a conserved way, suggesting that other molecules may bind similarly (Dickerhoff *et al.* 2021b). Taken together, the docking results, along with the fluorescent quenching measurements, suggest that the CX-5461 preferentially binds to the 5'-end but also can bind to the 3'-end of the c-*MYC* G4s structures.

### CX-5461 is a G4s stabilizer characterized by single-molecule magnetic tweezers

**Figure 2:**
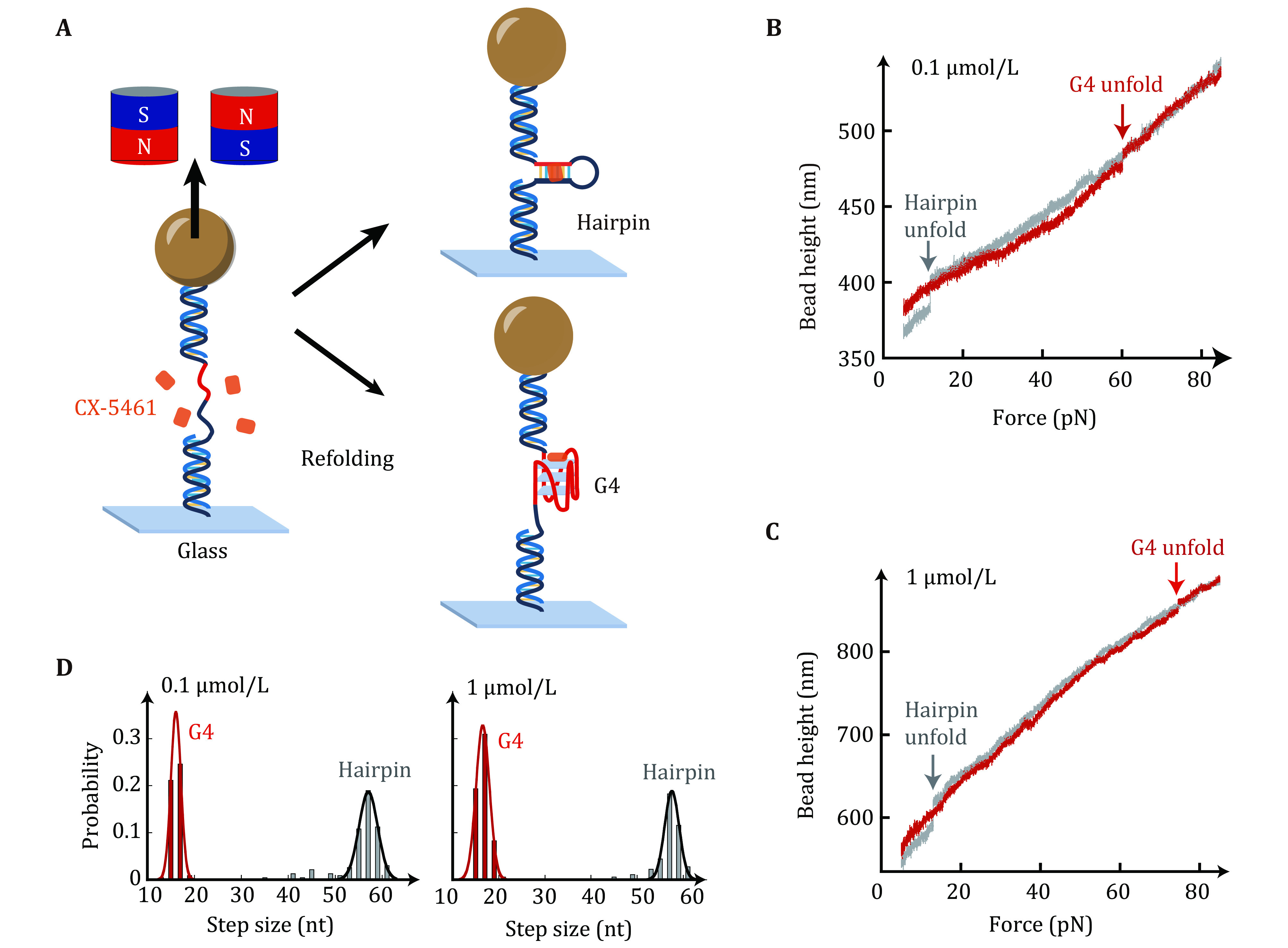
Single-molecule magnetic tweezers measurements for unfolding forces of c-*MYC* G4s in the presence of CX-5461. **A** Schematic of the experimental setup. A G4-forming sequence (red) and the complementary strand (black) spaced with a 20 nt thymine loop (Myc-20T) are ligated with two dsDNA handles (489 bp and 609 bp). The DNA construct is attached to a paramagnetic bead through biotin-streptavidin and a coverslip through a covalent cross-linker at each end. **B**,**C** The overlap of the force-extension curve of MYC-hairpin (grey) and MYC-G4 (red) in the presence of 0.1 µmol/L (**B**) and 1 µmol/L (**C**) CX-5461. **D** Unfolding step size analysis of Myc-20T G4s in the presence of 0.1 µmol/L CX-5461 (*n* = 232, left panel) and 1 µmol/L CX-5461 (*n* = 181, right panel). Data were fitted with Gaussian distribution

The impacts of CX-5461 on the unfolding forces of c-*MYC* G4s were characterized using single-molecule magnetic tweezers to unfold a short dsDNA hairpin that contains a c-*MYC* promoter G4 forming sequences and complementary strand (Fig. 2A) (Zhang *et al.* 2024). The Myc-hairpin DNA was ligated with two double-stranded DNA handles (489 bp and 609 bp) and was tethered between a streptavidin-coated paramagnetic bead and a coverslip. Force was applied to the tethered DNA by a pair of magnets and the bead height was monitored based on the diffraction pattern of the image of the beads. Figures 2B and 2C show the overlap of two typical force-bead height curves obtained by the force-ramp experiments using a constant loading rate of 2 pN/s from 5 pN to 90 pN. The sudden extension jumps appearing in force-ramp cycles indicate the unfolding transition of the hairpin (grey) or G4 structures (red), which can be distinguished from the unfolding step sizes as the hairpin and G4 structures are composed of 56 nt and 16 nt, respectively. Subsequently, the force jumps to 5 pN for 60 s to allow the refolding of hairpin or G4 structures. The unfolded ssDNA was held at 5 pN for refolding because force can increase the formation probability of G4s instead of the hairpin.

The Myc-20T hairpin construct was used because the CX-5461 intercalative binding into dsDNA handles can cause significant fluctuations in dsDNA extension (Liu *et al.* 2023). The intercalative binding of CX-5461 to dsDNA handle disrupts the typical overstretching transition of dsDNA usually observed at ~65 pN (Backer *et al.* 2019). The large unfolding step sizes of the hairpin (Figs. 2B and 2C, grey) can be used as a reference curve for detecting the G4s unfolding signal. Figure 2D shows the unfolding step size distribution of 232 stretching cycles from six independent Myc-hairpin molecules measured in 0.1 µmol/L CX-5461. A total of 109 unfolding events exhibited an unfolding step size of 16 ± 1 nt (average ± SD), while other 123 unfolding events displayed an unfolding step size of 56 ± 4 nt (average ± SD). These step sizes are consistent with the G4s and hairpin structures, respectively. In the presence of 1 µmol/L CX-5461 (right panel), the distribution of unfolding step sizes again revealed two peaks of 17 ± 1 nt (*n* = 107) and 57 ± 3 nt (*n* = 74), which are consistent with G4s and hairpin structures, respectively.

### Unfolding force distributions of G4 in the presence of CX-5461 represent different mechanical stability of 1:1 and 2:1 complexes

The unfolding force distribution $ {p}_{{\mathrm{unfold}}}\left(f\right) $ could be obtained by repeating the force-ramp stretching cycles and recording the forces at which unfolding events occurred. We next analyze the unfolding rates of CX-5461-G4 complexes by analyzing the unfolding force distributions using a Bell-Arrhenius model (Bell 1978), which described the force-dependent unfolding rate as:

1 $ {k}_{u}\left(f\right)=A\mathrm{exp}\left(\dfrac{-(\Delta {G}^{\text{‡}}- \Delta {x}_{u}f)}{{k}_{B}T}\right){=k}_{u}^{0}\mathrm{e}\mathrm{x}\mathrm{p}\left(\dfrac{\Delta {x}_{u}f}{{k}_{B}T}\right) , $

where $ {k}_{u}^{0} $ is the zero-force unfolding rate, and $ \Delta {x}_{u} $ is the distance between the folded state and the transition state. The zero-force unfolding rate is determined by $ {k}_{u}^{0}=A\mathrm{exp}\left(\dfrac{-\Delta {G}^{\text{‡}}}{{k}_{B}T}\right) $, where *A* is a prefactor. $ \Delta {G}^{\text{‡}} $ is the activation energy for G4s unfolding. The Bell-Arrhenius model predicts an unfolding force distribution with a single force peak $ {p}_{\mathrm{u}\mathrm{n}\mathrm{f}\mathrm{o}\mathrm{l}\mathrm{d}}^{\mathrm{B}\mathrm{e}\mathrm{l}\mathrm{l}}\left(f\right) $ as：

2 $ {p}_{\mathrm{u}\mathrm{n}\mathrm{f}\mathrm{o}\mathrm{l}\mathrm{d}}^{\mathrm{B}\mathrm{e}\mathrm{l}\mathrm{l}}\left(f\right)=\dfrac{{k}_{u}^{0}}{r}\mathrm{e}\mathrm{x}\mathrm{p}\left\{\dfrac{\Delta {x}_{u}f}{{k}_{B}T}+\dfrac{{k}_{B}T{k}_{u}^{0}}{\Delta {x}_{u}r}\left[1-\mathrm{exp}\left(\dfrac{\Delta {x}_{u}f}{{k}_{B}T}\right)\right]\right\} , $

where $ r $ is the loading rate (Evans and Ritchie 1997). We previously reported the single-peak unfolding force distribution of c-*MYC* G4s (Myc2345) at 55 pN and the best fitting to Eq. 2 determined $ {k}_{u}^{0}= (2.6 \pm 0.6)\times {10}^{-6} $ s^−1^ and $ \Delta {x}_{u}= 0.77 \pm 0.02 $ nm (You *et al.* 2015).

In the absence of CX-5461, the single-molecule force spectroscopy of c-*MYC* G4s measured using Myc-hairpin exhibited a single unfolding force peak at 56 ± 4 pN, consistent with previous reports using single-stranded c-*MYC* G4s sequence (You *et al.* 2015). The unfolding force increased upon CX-5461 binding (≥ 0.1 µmol/L). At 0.1 µmol/L CX-5461, two unfolding force peaks were observed. The lower peak (56 ± 5 pN) accounting for 67% of the population, likely represents the ligand-free G4s. The higher peak (73 ± 3 pN), representing the remaining 33%, likely corresponds to the 5'-bound CX-5461-G4 complex, supported by the *K*_d_ obtained from the fluorescent quenching assay. By fitting the measured unfolding force distributions $ {p}_{\mathrm{u}\mathrm{n}\mathrm{f}\mathrm{o}\mathrm{l}\mathrm{d}}^{}\left(f\right) $ to a two-unfolding force species $ \alpha {p}_{\mathrm{m}\mathrm{a}\mathrm{j}\mathrm{o}\mathrm{r}}^{\mathrm{B}\mathrm{e}\mathrm{l}\mathrm{l}}\left(f\right)+\left(1-\alpha \right){p}_{\mathrm{m}\mathrm{i}\mathrm{n}\mathrm{o}\mathrm{r}}^{\mathrm{B}\mathrm{e}\mathrm{l}\mathrm{l}}\left(f\right) $, we determined the fraction of each population and their zero-force unfolding rates $ {k}_{u}^{0} $ (Table 1). The global fitting employed three free parameters $ {k}_{u1}^{0} $, $ {k}_{u2}^{0} $, and $ \alpha $. A fixed $ \Delta {x}_{u} $ was used for both species based on our previously measured $ \Delta {x}_{u} $ for c-MYC G4s (You *et al.* 2015) and multiple single-molecule studies also suggest that G4s typically exhibit a short unfolding transition distance (<2 nm) (Cheng *et al.* 2020). The $ {k}_{u}^{0} $ for unbound and 5'-end bound states were estimated to be (2.3 ± 0.1) × 10^−7^ s^−1^ and (1.6 ± 0.2) × 10^−8^ s^−1^ respectively.

**Table 1:** Unfolding forces and zero-force unfolding rates of CX-5461-G4 complexes

Ligands concentration (µmol/L)	0:1	1:1	2:1	$ {\mathit{k}}_{\mathit{u}(0:1)}^{0} $ (s^−1^)	$ {\mathit{k}}_{\mathit{u}\left(1:1\right)}^{0} $ (s^−1^)	$ {\mathit{k}}_{\mathit{u}(1:2)}^{0} $ (s^−1^)
0.1	67% 59 ± 5 pN	33% 73 ± 3 pN		(2.3 ± 0.1) × 10^−7^	(1.6 ± 0.2) × 10^−8^	
1		74% 69 ± 5 pN	26% 84 ± 3 pN		(2.3 ± 0.2) × 10^−8^	(7.1 ± 1.5) × 10^−10^
2		44% 67 ± 5 pN	56% 79 ± 6 pN		(3.4 ± 0.9) × 10^−8^	(2.1 ± 0.4) × 10^−9^

**Figure 3:**
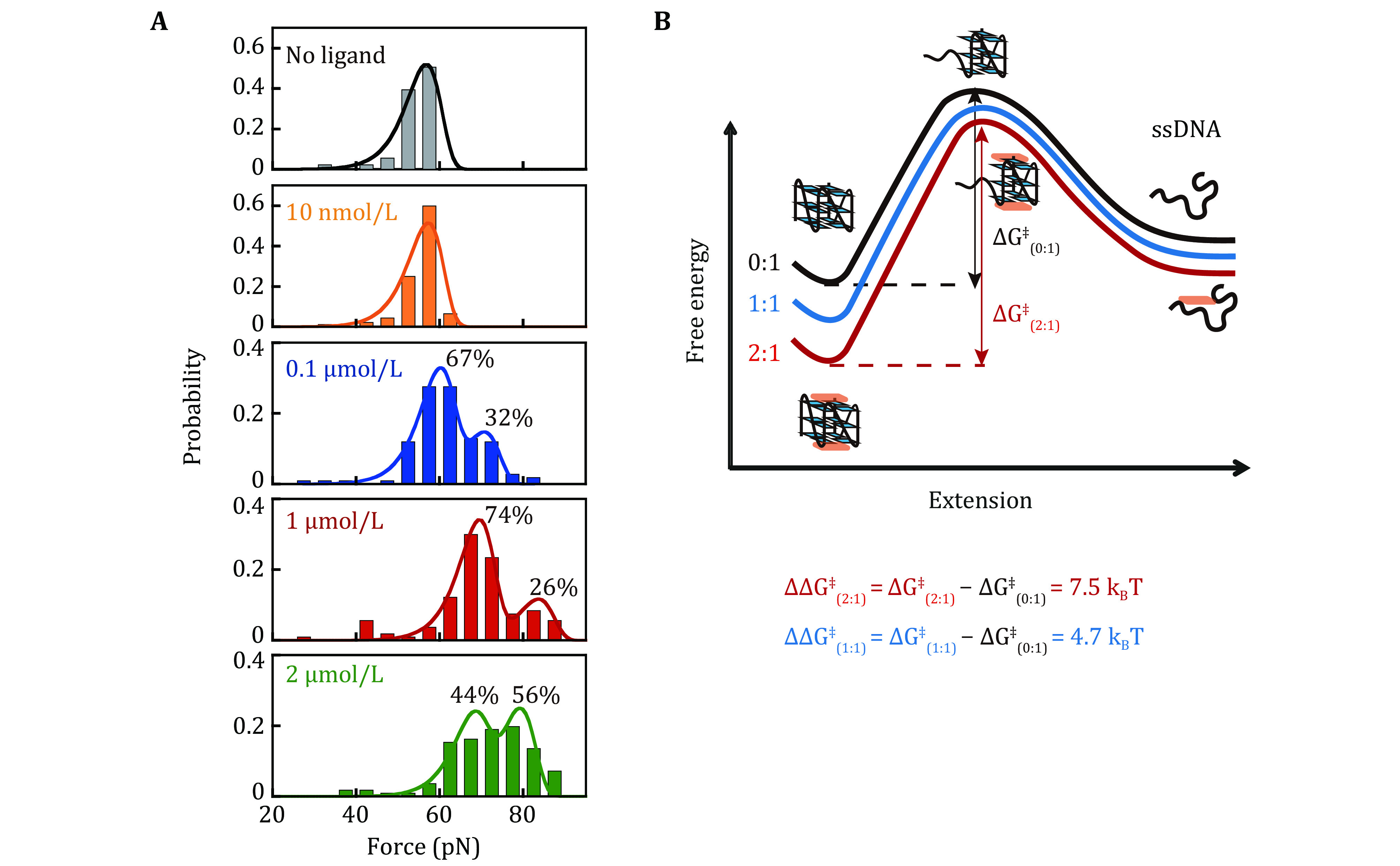
Unfolding force distribution of c-*MYC* G4s in the presence of CX-5461. **A** Unfolding force distributions of c-MYC G4s in the absence and presence of different concentrations of CX-5461. The data obtained at 0 nmol/L (grey) and 10 nmol/L CX-5461 (yellow) were fitted with a single peak using the Bell-Arrhenius model (Eq. 2). The data obtained at 0.1 μmol/L (blue), 1 µmol/L (red), and 2 µmol/L (green) CX-5461 were fitted with two peaks using the Bell-Arrhenius model. **B** Energy landscape reconstruction. The change of activation energy was calculated using $ \Delta {\mathit{G}}^{\text{‡}} $ and using $ {\mathit{k}}_{\mathit{u}}^{0}=\mathit{A}\mathbf{exp}\left(\dfrac{-\Delta {\mathit{G}}^{\text{‡}}}{{\mathit{k}}_{\mathit{B}}\mathit{T}}\right) $ assuming that ligands do not significantly change the prefactor A

As the CX-5461 concentration increased to 1 and 2 µmol/L (Fig. 3A), the unfolding force peaks shifted and the ~55 pN unliganded population disappeared. At 1 µmol/L, the lower peak (69 ± 5 pN) represented 74% of the population, while the higher peak (84 ± 3 pN) represented 26%. At 2 µmol/L, the lower peak (67 ± 5 pN) represented 44% of the population, while a higher peak (79 ± 6 pN) represented 56%. The corresponding zero-force unfolding rates determined by fitting the distributions were summarized in Table 1. Combining the results from the fluorescent quenching assay, molecular docking, and previously reported ligand-c-*MYC* G4 structures, we assigned the ~70 pN peak to the 1:1 complex where CX-5461 binds at the G4’s 5'-end. The ~80 pN peak likely corresponds to the 2:1 complex, where CX-5461 occupies both the 5’- and 3’-end. The average zero-force unfolding rates of 1:1 and 2:1 complexes were estimated to be (2.4 ± 0.9) × 10^−8^ s^−1^ and (1.4 ± 1.0) × 10^−9^ s^−1^, respectively.

## DISCUSSION

### *K*_d_ and binding energies of CX-5461 to 5'- and 3'-end of c-*MYC* G4s determined using unfolding force distributions

The binding free energy of CX-5461 to 5' and 3' can be also estimated based on the unfolding force distributions measured at different ligands concentrations. According to Boltzmann distribution, the probability of finding the molecule in a state *i* that has energy *Ui* is

3 $ {P}_{i}=\dfrac{1}{Z}{e}^{-{U}_{i}/{k}_{B}T}=\dfrac{{e}^{-{u}_{i}/{k}_{B}T}}{\sum {e}^{-{u}_{i}/{k}_{B}T}} , $

where Z is the partition function. Use the ligand free G4s as a control *U*_1_ = 0 (no ligand), the energy of G4s bound with ligand can be determined by $ {U}_{2}=-{k}_{\mathrm{B}}T\,\mathrm{l}\mathrm{n}\dfrac{C}{{{K}_{d}}_{1}} $ (1:1, 5'-end bound state), $ {U}_{3}=-{k}_{\mathrm{B}}T\left(\mathrm{l}\mathrm{n}\dfrac{C}{{{K}_{d}}_{1}}+\mathrm{l}\mathrm{n}\dfrac{C}{{{K}_{d}}_{2}}\right) = -{k}_{\mathrm{B}}T\,\mathrm{l}\mathrm{n}\dfrac{{C}^{2}}{{{K}_{d}}_{2}*{{K}_{d}}_{1}} $ (2:1, 5'-end and 3'-end bound state), $ {U}_{4}=-{k}_{\mathrm{B}}T\,\mathrm{l}\mathrm{n}\dfrac{C}{{{K}_{d}}_{2}} $ (1:1, 3'-end bound state), where $ {{K}_{d}}_{1} $ is the dissociation constant of 5’-end bound state, $ {{K}_{d}}_{2} $ is the dissociation constant of 3'-end bound state. The probability of different ligand-bound states can be described as follows:

4 $ \left\{\begin{aligned} &
{P}_{1}=\dfrac{1}{1+\dfrac{C}{{{K}_{d}}_{1}}+\dfrac{{C}^{2}}{{{K}_{d}}_{2}*{{K}_{d}}_{1}}+\dfrac{C}{{{K}_{d}}_{2}}}, \\&
{P}_{2}=\dfrac{\dfrac{C}{{{K}_{d}}_{1}}}{1+\dfrac{C}{{{K}_{d}}_{1}}+\dfrac{{C}^{2}}{{{K}_{d}}_{2}*{{K}_{d}}_{1}}+\dfrac{C}{{{K}_{d}}_{2}}} ,\\&
{P}_{3}=\dfrac{\dfrac{{C}^{2}}{{{K}_{d}}_{2}*{{K}_{d}}_{1}}}{1+\dfrac{C}{{{K}_{d}}_{1}}+\dfrac{{C}^{2}}{{{K}_{d}}_{2}*{{K}_{d}}_{1}}+\dfrac{C}{{{K}_{d}}_{2}}} ,\\&
{P}_{4}=\dfrac{\dfrac{C}{{{K}_{d}}_{2}}}{1+\dfrac{C}{{{K}_{d}}_{1}}+\dfrac{{C}^{2}}{{{K}_{d}}_{2}*{{K}_{d}}_{1}}+\dfrac{C}{{{K}_{d}}_{2}}} .
\end{aligned}\right. $

Rearrange the equation, we obtained $ \dfrac{{P}_{2}}{{P}_{1}}=\dfrac{C}{{{K}_{d}}_{1}} $, $ \dfrac{{P}_{3}}{{P}_{2}}=\dfrac{C}{{{K}_{d}}_{2}} $, where the ratio of two probability can be determined by the fraction of unfolding force peak. Because $ {{K}_{d}}_{2} $>> $ {{K}_{d}}_{1} $, at low concentrations of CX-5461, the two main peaks likely represent no liganded G4s (P1) and 5'-end bound state (P2). Based on the observed fractions of 67% and 32% at 0.1 µmol/L CX-5461, we determined $ {{K}_{d}}_{1} $ = 0.2 µmol/L for 5'-end binding, corresponding a binding free energy of −15.4*k*_B_*T*. At high concentration (*e*.*g*., 1 µmol/L CX-5461), the major two peaks likely represent the 5'-end bound state (P2) and 2:1 CX-5461-G4 complex (P3), with fraction of 74% and 26%, respectively. This suggests $ {{K}_{d}}_{2} $ = 2.8 µmol/L for 3'-end binding, corresponding to a binding free energy of –12.8*k*_B_*T*. The *K*_d_ measured using single-molecule force spectroscopy were higher than that obtained from the fluorescent quenching assay (Fig. 1). This discrepancy arises because force spectroscopy detects only interactions that significantly stabilize the G4 structures. In contrast, the fluorescence quenching assay might capture both specific binding events that stabilize the G4s and non-specific interactions between the ligand and G4s that do not affect G4s stability.

### The effects of CX-5461 on the activation energy of c-*MYC* G4s unfolding estimated from $ {\mathit{k}}_{\mathit{u}}^{0} $

By fitting the unfolding force distribution with the Bell-Arrhenius model, we obtain the zero-force unfolding rates of 0:1, 1:1, and 2:1 complex (Table 1). These rates fall in the range of 10^–7^ s^–1^, 10^–8^ s^–1^, and 10^–9^ s^–1^ respectively. Analyzing the zero-force unfolding rates $ {k}_{u}^{0} $ of different species allowed us to estimate the changes in activation energy $ \Delta {G}^{\text{‡}} $ for unfolding using the equation $ {k}_{u}^{0}=A\mathrm{exp}\left(\dfrac{-\Delta {G}^{\text{‡}}}{{k}_{B}T}\right) $. While ligand binding can alter the unfolding pathway, leading to a higher activation energy for unfolding, multiple previous force spectroscopy studies of various G4 structures suggest a short unfolding transition distance (< 2 nm) (Cheng *et al.* 2021). This implies that the transition state shares a similar structure with the folded G4s. Based on this, it is reasonable to assume that CX-5461 binding likely doesn’t significantly change the unfolding pathway, and the pre-factor A remains relatively constant. Therefore, the observed decrease in $ {k}_{u}^{0} $ primarily reflects the changes in activation energy $ \Delta {G}^{\text{‡}} $ for unfolding. Using $ \Delta {\Delta G}^{\text{‡}}={-k}_{B}T\,\mathrm{ln}\dfrac{{k}_{u(i:1)}^{0}}{{k}_{u(0:1)}^{0}} $, we calculated the activation energies change $ {\Delta \Delta G}_{(1:1)}^{\text{‡}} $ = 4.7$ {k}_{B}T $ and $ {\Delta \Delta G}_{(2:1)}^{\text{‡}} $ = 7.5$ {k}_{B}T $ for the 1:1 and 2:1 complex, respectively. The increased activation energy upon CX-5461 binding to G4s suggests that the CX-5461 has a stronger binding affinity to the folded G4s structure compared to the transition state. The binding of CX-5461 at both the 5'- and 3'-end of G4s further increased the unfolding energy barrier than only bound to the 5'-end of G4s.

It is important to note that reconstructing the free energy landscape of G4s in the presence of ligands requires careful consideration as the effects of ligands on the c-*MYC* G4s folding/unfolding pathway can be complicated. Previous studies have reported the existence of folding intermediates for c-*MYC* G4s (Gray *et al.* 2019). Small ligands, such as PDS, can result in even lower unfolding force peaks observed in single-molecule force spectroscopy experiments (Zhang *et al.* 2024). Determining the stoichiometries, binding modes, and structural diversity of G4s remains important for resolving the effects of ligands on G4s folding/unfolding dynamics.

## CONCLUSION

This work employed single-molecule magnetic tweezers to quantify the unfolding force distributions of c-*MYC* G4s in the presence of different concentrations of CX-5461. This analysis allowed us to quantify the zero-force unfolding rates of distinct bound states within the CX-5461-G4 complexes. By combing with a fluorescence quenching assay and molecular docking simulations of previously reported ligand-G4 complexes, we assigned the unfolding force peaks to 1:1 and 2:1 complex, where CX-5461 interacts with 5'-end or both the 5'- and 3'-end of c-*MYC* G4. Furthermore, by fitting the unfolding force distributions with the Bell-Arrhenius model, we obtain the zero-force unfolding rates of 1:1, and 2:1 CX-5461-G4 complexes be (2.4 ± 0.9) × 10^−8^ s^−1^ and (1.4 ± 1.0) × 10^−9^ s^−1^, respectively. The unfolding force distribution can be used to calculate the binding free energies of CX-5461-G4 complexes and reconstruct the unfolding energy landscape. The methods presented in this study are applicable to a broad spectrum of ligand-G4 interactions, offering valuable insights into the design of G4-targeting ligands for cancer treatments.

## MATERIALS AND METHODS

### Materials and reagents

CX-5461 was purchased from TargetMol (USA) and stored at –80 °C in DMSO before use. Myc-hairpin DNA: 5’TAGGGTGGGGAGGGTGGGTT(T)_20_AACCCACCCTCCCCACCCTA was purchased from Sangon Biotech Co., Ltd (China). The Myc-hairpin containing c-*MYC* G4s forming sequences and the complementary strand were annealed with two flank sequences and then ligated with a BstX1 (Thermo Fisher Scientific, USA) digested dsDNA handles as previously described (Zhang *et al.* 2024). The 5′-thiol labeled 489 bp and 5′-biotin labeled 609 bp dsDNA handles were generated via PCR amplification using 5’-thiol and 5’-biotin primers (Genewiz, China) and DreamTaq DNA polymerase (Thermo Fisher Scientific, USA) using lambda phage DNA (Thermo Fisher Scientific, USA) as the template.

### Single-molecule magnetic tweezers

Magnetic tweezers experiments were performed with BioPSI magnetic tweezers (BioPSI, Singapore). DNA constructs were covalently tethered to a (3-Aminopropyl) Triethoxysilane (Cool Chemical Technology, China) functionalized coverslip via a Sulfo-SMCC (Hunan Hua Teng Pharmaceutical, China) cross-linker. To block nonspecific interactions, bovine serum albumin (BSA) buffer (10 mg/mL BSA, 1 mmol/L 2-mercaptoethanol, 1× PBS, pH 7.4) was introduced into the flow chamber for overnight incubation. Streptavidin-coated paramagnetic beads (Dynal M280, 2.8 μm diameter, Thermo Fisher Scientific, USA) were introduced to attach the 5' biotin-labeled end of DNA. All single-molecule experiments were conducted at room temperature (20–23 °C) in a working buffer (10 mmol/L Tris-HCl, 100 mmol/L KCl, pH 8.0).

### Fluorescence quenching assay and *K_d_* determination

Oligonucleotides labeled with 5'-FAM (excitation 490 nm, emission 520 nm) or 3'-FAM were purchased from Genewiz. 10 nmol/L oligonucleotides and varying ligand concentrations were diluted in assay buffer (10 mmol/L Tris-HCl, pH 8.0, 10 mmol/L KCl, 90 mmol/L LiCl) at a final volume of 20 μL. The fluorescent intensity was measured at 25 °C for 100 s using a Qiagen 36-well RT-PCR instrument (Qiagen Rotor-Gene Q, Germany). The dissociation constant *K*_d_ was determined using a quartic equation instead of the Hill equation to minimize the effects of the depletion of the free ligand due to binding to G4s (Thordarson 2011).

5 $ {K}_{d}=\dfrac{{c}_{\mathrm{ligand}}{c}_{\mathrm{DNA}}}{{c}_{\mathrm{ligand-DNA}}}=\dfrac{({c}_{\mathrm{ligand}}^{0}-{c}_{\mathrm{ligand-DNA}})({c}_{\mathrm{DNA}}^{0}-{c}_{\mathrm{ligand-DNA}})}{{c}_{\mathrm{ligand-DNA}}} , $

in which $ {c}_{\mathrm{ligand}} $ and $ {c}_{\mathrm{DNA}} $ are the concentrations of free ligand and substrate (DNA), respectively, and $ {c}_{\mathrm{ligand-DNA}} $ is the concentration of the ligand−DNA complex. The concentrations of total ligands and total substrate (DNA) are denoted by $ {c}_{\mathrm{ligand}}^{0} $ and $ {c}_{\mathrm{DNA}}^{0} $, respectively. According to the mass balance,

6 $ \left\{\begin{aligned} &
{c}_{\mathrm{ligand}}^{0}={c}_{\mathrm{ligand}}+{c}_{\mathrm{ligand-DNA}},\\& {c}_{\mathrm{DNA}}^{0}={c}_{\mathrm{DNA}}+{c}_{\mathrm{ligand-DNA}}. 
\end{aligned}\right.$

Based on Eqs. 5 and 6, Eq. 7 containing only the total concentration of ligand and DNA can be derived:

7 \begin{equation*}\begin{split} &
{\left({c}_{\mathrm{ligand-DNA}}\right)}^{2}-\left({c}_{\mathrm{ligand}}^{0}+{c}_{\mathrm{DNA}}^{0}+{K}_{d}\right){c}_{\mathrm{ligand-DNA}}\\&
+{c}_{\mathrm{ligand}}^{0}{c}_{\mathrm{DNA}}^{0}=0.
\end{split}\end{equation*}

Equation 7 has only one relevant real solution (Eq. 8):

8 \begin{equation*}\begin{split} 
{c}_{\mathrm{ligand-DNA}}=\;&\dfrac{1}{2}\Bigg[\left({c}_{\mathrm{ligand}}^{0}+{c}_{\mathrm{DNA}}^{0}+{K}_{d}\right)\\&
-\sqrt{{\left({c}_{\mathrm{ligand}}^{0}+{c}_{\mathrm{DNA}}^{0}+{K}_{d}\right)}^{2}-4{c}_{\mathrm{ligand}}^{0}{c}_{\mathrm{DNA}}^{0}}\Bigg] .
\end{split}\end{equation*}

The bound fraction of the substrate molecules $ \alpha =\dfrac{{c}_{\mathrm{ligand-DNA}}}{{c}_{\mathrm{DNA}}^{0}} $,

9 \begin{equation*}\begin{split} 
\alpha =\;&\dfrac{1}{2{c}_{\mathrm{DNA}}^{0}}\Bigg[\left({c}_{\mathrm{ligand}}^{0}+{c}_{\mathrm{DNA}}^{0}+{K}_{d}\right)\\&
-\sqrt{{\left({c}_{\mathrm{ligand}}^{0}+{c}_{\mathrm{DNA}}^{0}+{K}_{d}\right)}^{2}-4{c}_{\mathrm{ligand}}^{0}{c}_{\mathrm{DNA}}^{0}}\Bigg] .
\end{split}\end{equation*}

Equation 9 was used for fitting the bound fraction $ \alpha $ based on the fluorescence intensity change, $ \alpha =\dfrac{\Delta {I}_{c}}{\Delta {I}_{\mathrm{max}}} $ at varying ligand concentrations to determine the *K*_d_. $ \Delta {I}_{\mathrm{max}} $ represents the maximum fluorescent intensity change and $ \Delta {I}_{c} $ represent the fluorescent intensity change at a total ligand concentration of $ {c}_{\mathrm{ligand}}^{0} $. We note that Eqs. 5–9 were derived assuming a 1:1 ligand-G4 binding stoichiometry. Because *K*_d_ at 5’- and 3’-end significantly different, thus allowed us to use this model to determine *K*_d_ at 5’-end. For *K*_d_ at the 3’-end, where *K*_d_ is much higher than the concentration of FAM-labeled oligo (20 nmol/L), thus minimized the effects caused by depletion of free ligand due to binding to G4s.

### Molecular docking and molecular dynamics simulation

The NMR structure of the c-*MYC* G4 in complex with a carbazole derivative BMVC was used for semiflexible global docking (2:1 complex, PDB: 6O2I) (Liu *et al.* 2019). Semiflexible global docking with a Lamarckian Genetic Algorithm was carried out using Autodock 4.2.6 and AutoTools 1.5.7 (graphical user interface). The lowest binding energy conformations for the 5’-end complex and 3’-end complex were further validated using molecular dynamic simulation using the Gromacs-2019.6 simulation package and Amber parmbsc1 force field (Ivani *et al.* 2016). A solute box distance of 2.5 nm was defined, setting the minimum distance of 5.0 nm between any two periodic images of a G4s-ligand complex. The TIP3P water molecules soaked the G4-ligand complex model, and system neutralization was carried out using 0.15 mol/L potassium chloride. The ion parameters were from the default ones for the Amber force field. Equilibration of the systems was performed for 125 ps under an NVT ensemble. A 100 ns MD runs were completed and the RMSD values of all atoms of the G4s-ligand complexes from the average structure were analyzed.

## Conflict of interest

Hui Peng, Yashuo Zhang, Qun Luo, Xinyu Wang and Huijuan You declare that they have no conflict of interest.
